# Miscellaneous Notices

**Published:** 1849-10

**Authors:** 


[*Dental News Letter.*

### Miscellaneous Notices.

*Mr. Evans' New Amalgam.*—We published, in the last number of the Journal, a letter from Mr. Evans, explanatory of his views on the subject of filling teeth with amalgam; and the following is another letter which we received from him upon the same subject—a duplicate of which was recently published in the Dental News Letter. No one, we think, after reading this letter, will regard Mr. E. as an advocate for the use of amalgam in filling teeth. The result of his experience, thus far, even with his new amalgam, which is supposed to be less objectionable than the amalgam heretofore employed, is not as satisfactory as the advocates for the use of this article could have desired.—*Balt. Ed.*

Paris, *Sept.* 20*th*, 1849.

Dr. C. A. Harris:

*Dear Sir*:—In compliance with a promise given to my professional brethren: I should like to have inserted in the "American Journal of Dental Science," if space permits, my present views in regard to the preparation of tin and cadmium, an account of which I published in the London Lancet and London Medical Gazette. It appears to have been inferred from my remarks in these journals, that I am an advocate of the practice of filling teeth with amalgams. This is an honor to which I have not the slightest claim whatever—and I must therefore leave it entirely to the enjoyment of those who are justly entitled to it. The fact is well known to my friends on this side of the Atlantic, as well as in America, that I have always been strongly opposed to the use of any other material for filling teeth than gold; and in my own practice I have never made use of any other. My predominant opinion has always been decidedly in favor of gold, notwithstanding I have, in common with others, experimented, with a view, on my part, of testing the merits of substances designed to be used in a plastic state. From my experiments with the preparation alluded to, I discovered it possessed qualities that other amalgams did not. It preserves its color better than the other mercurial preparations I am acquainted with. It also has the advantage of becoming a tough ductile substance, susceptible of being cut or burnished like a piece of tin. In addition, the cadmium seems to completely absorb the mercury in the process of crystallization. From these circumstances, it was thought to possess important advantages over any of the substances hitherto employed.

There is, in my opinion, a very great objection to all the combined metals, arising from the fact that they are all promoters of galvanic action. This objection is a very serious one in a substancc employed for the purpose of filling teeth. Upon removing some of this filling, which appeared perfectly tight, and which was unchanged in its color upon the exterior surface, I have found that beneath the metal a deep yellow hue had made its appearance, penetrating for some distance into the bone of the tooth. This phenomenon does not present itself until after the lapse of considerable time, and appears much more striking in some cases than in others. Whether this effect is to be ascribed to galvanic agency yet remains to be determined. Mr. Faraday observes, (see Turner's Chemistry, 6th Am. edition, p. 399,) that an alloy of steel with one-hundredth part of its weight of platina, dissolves with effervescence in diluted sulphuric acid, so weak as scarcely to act on common steel; a fact which he accounts for by ascribing it to the steel being rendered positive by the presence of the platina.

I have watched the effects produced by the application of this preparation, and the result of my observation has been such as I have stated above. I am now engaged in investigating the subject of the different amalgams that have been in use, in reference to their influence upon the general health; and I hope soon to be able to give the opinion of some of the highest medical authorities in Europe, upon a question which has elicited so much discussion, and which, in reality, is one of the greatest importance.

In giving publicity to this preparation, the object was not simply to make known the result of my experiments, but also to invite the attention of the profession to a subject so peculiarly important as that of discovering a material to be used *in those cases in which the circumstances might seem to indicate the expediency of employing a soft filling*, and to take the place of the compositions deemed objectionable, already in use. Experience, the great test in such matters, has furnished a result not so favorable as was anticipated by many members of the profession, who had expressed their decided approbation of this preparation, and who seemed perfectly confident that it was destined to fill a great desideratum which had so long been felt.

Whatever may be the hopes of others regarding this preparation, I have never considered it a substitute for gold. I have regarded it as a mere expedient, to be resorted to only in *peculiar* cases.

In regard to its influence upon the general health, in the opinion of those whose authority is entitled to weight, it is not injurious in this respect. *I cannot, however, refrain from stating it as my deliberate opinion, that all*

*operations in which amalgams are employed, are merely temporary in their nature, and that any tooth that can be filled in a* PROPER *manner with gold, can be effectually and permanently saved only by this means.*

This is the opinion I have always entertained, and I adhere to it at the present moment, with *undiminished* confidence.

Yours, respectfully,

THOS. W. EVANS.

15 Rue de la Paix, Paris.

*A New Dentist's Lathe.*—We have used the lathe described in the following communication, and find it to work well; indeed, we think it superior to any we have ever seen —*Balt. Ed.*

Dr. Harris:

*Dear Sir:*—In compliance with your request, I send a brief description of the lathe which you received a few days since—though, without a drawing and figures of reference, scarcely any idea can be conveyed of its form and appearance. The arbor upon which the grinding-stones are mounted has firmly placed upon it a heavy fly wheel, revolving between two columns, so constructed that any wearing of the centre or boxes may immediately be remedied by starting the set-screws attached to each. To one column is appended the crank and driving wheel, which, together with its pinion, is made with diagonal leaves, (technically, spiral-gearing,) giving three revolutions of the fly wheel to one of the crank, and producing a sufficiently swift, strong and steady motion for any purpose to which such a machine can be applied in our profession.

The base from which the columns spring, is made hollow beneath, for the reception of slips of thick woollen cloth or gum-elastic, previously to securing it upon the table, for the purpose of breaking the vibration of sound, rendering the machine much stiller in motion than it would otherwise be. The rest for the hand is attached to the base, turning upon a center, and held in position by a binding-screw, nearer or farther from the running arbor, as the nature of the work may require.

Its advantages over a common lathe are—that while its compactness and beauty render it a perfectly appropriate article for the operating room, it will do all, in the mechanical department, which can be done by an ordinary lathe—occupying only a few square inches of space.

Having used it, both in my office and laboratory, I can speak confidently of the amount of work which it will perform. I have taken measures for its registration at Washington, and shall soon be able to furnish them to the profession, of three grades of finish—the running parts substantially the same in all. I am, respectfully, your ob't servant,

M. PRATT.

77 Lexington-st., *Nov.* 13, 1849.

The article to which the following note refers, was not received in time for publication in the present number of the Journal. It shall appear, however, in our next.—*Balt. Ed.*

Prof. C. A. Harris:

*Dear Sir:*—Having recently seen a number of articles in the southern newspapers, claiming for Mr. C. H. Dubs the priority of invention of the Compound Screw Forceps, over Dr. S. P. Hullihen, I enclose you an article for the American Journal of Dental Science, embracing *facts* which must forever set the question at rest.

I hope you may be able to insert the enclosed paper in the number of the Journal which is now in progress of publication, that misrepresentation of facts may be early corrected, both in justice to Dr. Hullihen, who has refrained from any participation in the discussion—and Mr. Dubs, as a man of honorable feeling, and who has assumed from mistake, or been placed by friends, not in a questionable, but absolutely false position.

I am, respectfully, yours, &c.

38 N. Charles-st, Baltimore, *October 27th*, 1849. C. O. CONE.

*Singular Aberration in the Position of a Tooth.*—A few weeks since, a youth, about sixteen years of age, called upon us to have the right side of his upper jaw examined, which he stated had been sore and painful for one or two years. On looking into his mouth, the first thing that attracted our attention, was the absence of the second bicuspis. The gum, between the first bicuspis and the first molar, on the buccal surface of the alveolar border, was inflamed, swollen and of a purple red color, and just below the reflection of the mucous membrane upon the inner wall of the cheek, we discovered a fistulous opening. On introducing a probe, a hard substance was felt, a little below the malar bone. This we soon ascertained to be the crown of a tooth, which, after enlarging the fistulous opening, we with some difficulty extracted. It was the second bicuspis, and about one-fourth of the root had penetrated the outer wall of the maxillary sinus.—*Balt. Ed.*

*An Antagonizing Instrument for Artificial Teeth.*—We have received from Mr. Evans, of Paris, a very simple contrivance for antagonizing artificial teeth. With the exception of a plaster antagonizing model, it is superior to any we have seen. We reserve a description—or rather an engraving, which will be more satisfactory and better understood—for a future number of the Journal; but, in the mean time, beg to tender our thanks to Mr. E., for his kindness in sending it to us.—*Balt. Ed.*

***Fifth Annual Meeting of the Mississippi Valley Association of Dental Surgeons.***—The Society met, pursuant to adjournment, at Louisville, September 11th, 1849, the meeting being held in the Temperance Hall.

At 10 o'clock, A. M., the President, Dr. Goddard, took the chair, and called the meeting to order, when the following members were present: Drs. Goddard, Jas. Taylor, Leslie, Allen, Taft, Curtiss, Vaneman, Bonsall, Griffith and McCullom.

The President's address being called for, he arose and read a paper of great interest, which, on motion of Dr. Taylor, was accepted, and ordered to be published.

Dr. J. W. Bright, of Louisville, was elected to honorary membership.

On motion of Dr. Griffith, the chair appointed Drs. Taylor, Leslie and Curtiss a committee to examine the specimens of mineral teeth presented in competition for the society's premiums offered at the fourth annual meeting.

The corresponding secretary, (Dr. E. Taylor,) being prevented from attending by indisposition, sent in a report by the hands of Dr. Jas. Taylor, stating that he had notified the delinquent members of the society, as instructed by the society, but had not been favored with a response in any case, and that their names were, in consequence, omitted in the published list. He also gave notice, as instructed by the society, to all the manufacturers of mineral teeth that he could hear of, of the premium offered by the society, and respectfully submits the lots received for examination.

The Publishing Committee being called upon, reported various matters of interest in regard to the publication of the Dental Register of the West.

On motion, it was *Resolved,* That eight dollars additional be appropriated to meet the contingent expenses of the Register for the past year.

On motion of Dr. Bonsall, *Resolved,* That the chair appoint a committee of three, to consider the project of altering or amending the present mode of publishing the Register. Whereupon, the President appointed Drs. Taylor, Griffith and Allen said committee.

The Treasurer presented his annual report, which was read and referred to an auditing committee, consisting of Drs. Griffith, Vaneman and Curtiss.

Dr. E. Taylor, being about to retire from the profession, tendered his resignation of membership, which, on motion, the society accepted, but expressed its deep regret at the loss of so active a member.

Dr. Leslie moving, Dr. Griffith seconding, Dr. E. Taylor was unanimously elected an honorary member of the Society.

On motion, the Society adjourned to meet at 3, P. M.

*Afternoon Session.*—Society called to order by the President, who announced an address by Dr. Leslie to be now in order. Dr. L. occupied the attention of the Society for some time, in reading a paper on Metallurgy, which, on motion, was accepted, and ordered to be published.

The auditing committee reported that they found the Treasurer's account correct, and that there was left in his hands a balance of $69 60.

Drs. Griffith and Taft read addresses, which were, on motion, accepted.

On motion of Dr. Taylor, the following resolution was adopted:

*Resolved,* That this Society view with pleasure the success which has attended the recent plastic operation of our professional brother, Dr. S. P. Hullihen, of Wheeling, Va., on Miss M——, and that the skill exhibited by the Doctor, in that delicate and important operation, is worthy of our highest consideration and imitation.

On motion, the Society adjourned to meet in the office of Dr. Goddard, at 7½ o'clock.

*Evening Session.*—Society met pursuant to adjournment. The address of Dr. Curtiss being next in order, was read and accepted.

Dr. S. C. Gray, of Cincinnati, was proposed and elected to membership.

The remainder of the evening was spent by the Society in an interlocutory, embracing several principles and modes of practice, which proved so advantageous as to induce the Society to resolve they would have a repetition of the same at their next meeting.

On motion, Society adjourned, to meet at 9 o'clock, A. M., at the Hall of the Sons of Temperance.

*Second Day, 9 o'clock,* A. M.—Society met according to adjournment; meeting called to order by the President.

Minutes of first day read and approved.

The President announced the order of the day to be the reading and consideration of the report of the committee appointed at the fourth annual meeting of the Society, to report on the best mode of filling teeth. Dr. Jas. Taylor, from that committee, stated, that owing to the absence of two of its members, the committee had not been able to confer on the subject; but he had received from each of the absentees a communication, exhibiting their views, which he would read to the Society, in order to elicit discussion on this imporant point of practice. After reading and discussion, these papers were, upon motion, referred to the publishing committee, with instructions to publish an abstract of the same.

The committee to whom was referred the subject of altering or amending the present mode of publishing the Register, reported by recommending the continuance of its publication as at present conducted.

On motion, *Resolved,* That the Society do now go into an election of officers to serve for the coming year.

After balloting, the following gentlemen were declared duly elected: Jas. Taylor, *President.* John Allen, 1*st Vice-president.* Samuel Griffith, 2*d Vice-pres't.* A. M. Leslie, 3*d Vice-pres't.* W. H. Goddard, *Cor. Secretary.* John Allen, *Rec. Secretary.* J. Taft, B. T. Whitney, A. Berry, *Examining Committee.* S. P. Hullihen, G. L. Vaneman, J. G. Curtiss, *Exec. Com.* Jas. Taylor, W. H. Goddard, A. M. Leslie, *Pub. Com.*

On motion of Dr. Goddard, it was unanimously

*Resolved,* That this Society pledge itself to the Publishing Committee, to relieve it from any personal liability which its members may necessarily incur in the publication of the Register.

On motion, adjourned to meet at 3 o'clock, P. M.

*Afternoon Session.*—Society met according to adjournment.

The committee on mineral teeth being called upon, reported that they had performed the duty assigned them, and recommended the award of the gold medal for the lot marked A, and the silver one for that marked B.

The Society accepted the report, and, after due inspection of the specimens, awarded the premiums accordingly.

The President then announced Messrs. Jones, White & McCurdy to be the manufacturers of the specimen marked A, and Mr. James Alcock, manufacturer of that marked B.

On motion of Dr. Taylor, it was made the duty of the Treasurer to have prepared the gold and silver medals, with suitable device and inscriptions, expressive of the Society's object in awarding the same, and to have the medals presented to the successful competitors.

By resolution, it was made the duty of the Corresponding Secretary, to make known to Messrs. Jones, White & McCurdy, and Mr. James Alcock, the award just made by the Society.

Dr. Taft offered the following resolution, and moved its adoption, which carried unanimously:

*Resolved,* That the thanks of the Society be awarded to the Editors of the Register, for their untiring energy in conducting its publication.

On motion of Dr. Leslie, *Resolved,* That when we adjourn, we do so to meet the second Tuesday in September, 1850, at the lecture room of the Ohio College of Dental Surgery.

On motion of Dr. Goddard, two dollars were appropriated to pay the person in attendance at the Hall. Also,

*Resolved,* That the thanks of the Association be presented to the Trustees of the Sons of Tempernce Hall, for the use of their rooms.

The following gentlemen were appointed essayists for the year 1850: Dr. Whitney, on Professional Etiquette; Dr. H. McCullom, on Filling Teeth; Dr. Bonsall, on Alveolar Abscess; Dr. John Allen, on Artificial Gums; Dr. Vaneman, on Blow-Pipe; Dr. Taft, on Dentition; Dr. Gray, Connection of Chemistry and Dentistry.

On motion of Dr. Gray,

*Resolved,* That the thanks of this Society be tendered to Drs. Goddard and Griffith, for their generous hospitalities extended to the members during its present session.

On, motion, adjourned.

W. H. GODDARD, *President*

JOHN ALLEN, *Rec. Sec'y.*

[*Dent. Reg. of the West.*

